# Artificial neural network-based immune biomarker signature predicts pathological complete response to neoadjuvant chemotherapy in HER2-negative breast cancer

**DOI:** 10.3389/fonc.2026.1781380

**Published:** 2026-03-04

**Authors:** Yulong Zhang, Shitang Nong, Suai Deng, Qiyuan Su, Jiao Lu, Dalang Fang, Shihuan Qin, Yanfei Ma

**Affiliations:** 1 Department of Gland Surgery, Baise People’s Hospital, Baise, Guangxi, China; 2 Department of Gland Surgery, Affiliated Hospital of Youjiang Medical University for Nationalities, Key Laboratory of Tumor Molecular Pathology of Baise, Baise, Guangxi, China; 3 Department of Ultrasound, Affiliated Hospital of Youjiang Medical University for Nationalities, Baise, Guangxi, China

**Keywords:** artificial neural network, HER2-negative breast cancer, immune biomarkers, neoadjuvant chemotherapy, pathologic complete response

## Abstract

**Background:**

Neoadjuvant chemotherapy (NAC) is widely used in early-stage and locally advanced HER2-negative breast cancer, yet pathological complete response (pCR) is achieved in only a subset of patients. Reliable pretreatment biomarkers for predicting pCR are lacking, particularly for patients treated with standard anthracycline- and taxane-based regimens. Increasing evidence indicates that chemotherapy efficacy is closely linked to the tumor immune microenvironment, suggesting that immune-related molecular signatures may improve response prediction.

**Methods:**

A total of 2,385 pretreatment HER2-negative breast cancer patients from ten GEO cohorts were included. GSE194040 (n = 743) was used for training, and nine independent cohorts (n = 1,642) were used for external validation. Differential expression analysis was performed separately in hormone receptor positive and negative subgroups, and genes showing concordant regulation between pCR and non-pCR cases were identified. Weighted gene co-expression network analysis (WGCNA) was applied to detect pCR-associated gene modules. Immune-related genes curated from the ImmPort database were intersected with candidate genes, followed by feature selection using least absolute shrinkage and selection operator regression, random forest, and support vector machine recursive feature elimination. An artificial neural network (ANN) model was constructed based on overlapping features and evaluated using receiver operating characteristic analysis. Immune infiltration was estimated by CIBERSORT, and transcription factor, competing endogenous RNA, and drug enrichment analyses were performed. Key genes were further validated by quantitative real-time PCR in pretreatment tumor tissues.

**Results:**

Five immune-related genes (CCL2, CXCL10, CXCL13, HLA-E, and IGKV1D-8) were identified as robust predictors of pCR and used to build the ANN model. The model achieved an area under the curve of 0.858(95% CI: 0.829–0.888) in the training cohort and 0.773 (95% CI: 0.735–0.808) in the external validation cohorts, demonstrating s predictive performance across independent datasets. High expression of the five-gene signature was associated with increased infiltration of cytotoxic and antigen-presenting immune cells, consistent with an immune-activated tumor microenvironment, and was confirmed by qRT-PCR analysis.

**Conclusion:**

This study establishes a rigorously validated ANN-based immune gene signature for predicting response to neoadjuvant chemotherapy in HER2-negative breast cancer, providing a potential tool for pretreatment risk stratifictableation and individualized therapeutic decision-making.

## 1 Introduction

Neoadjuvant chemotherapy (NAC) is a standard treatment for patients with early-stage or locally advanced breast cancer. Its primary objectives are to reduce tumor burden, facilitate breast-conserving surgery, and enable early evaluation of therapeutic efficacy. Achieving a pathologic complete response (pCR) is strongly associated with improved event-free and overall survival (1). In recent years, multiple real-world studies and systematic reviews have demonstrated that the use of NAC has markedly increased among patients with operable or locally advanced breast cancer. Notably, in high-risk subtypes such as HER2-positive disease, NAC has gradually expanded from locally advanced to high-risk early-stage patients and has become one of the standard treatment strategies (2, 3). However, for patients with HER2-negative tumors, anthracycline- and taxane-based regimens remain the mainstay of NAC, and the pCR rate varies markedly according to hormone receptor (HR) status: approximately 20-40% of HR-negative patients achieve pCR, whereas the rate in HR-positive tumors is only around 10% (4–6). This discrepancy not only reflects intrinsic biological heterogeneity but also underscores the limitations of current therapeutic approaches. Unlike HER2-positive patients who benefit from targeted anti-HER2 agents such as trastuzumab and pertuzumab, HER2-negative patients lack effective molecularly targeted therapies and rely primarily on conventional cytotoxic chemotherapy. Given the substantial toxicities associated with chemotherapy—including myelosuppression, neurotoxicity, alopecia, and cardiotoxicity—patients with limited benefit may experience unnecessary treatment burden. Therefore, identifying reliable biomarkers capable of predicting pCR before treatment is of great clinical importance for guiding precision therapy, avoiding ineffective chemotherapy, and minimizing unnecessary adverse effects in HER2-negative breast cancer.

Currently, prediction of NAC response in breast cancer largely depends on tumor subtype and clinicopathological characteristics, but these indicators have limited accuracy. For example, although HR-positive/HER2-negative tumors generally have low pCR rates, a subset of these patients still responds favorably, suggesting underlying biological heterogeneity. Accumulating evidence indicates that the tumor immune microenvironment plays a pivotal role in modulating chemotherapy sensitivity. Multiple studies have shown that patients with high levels of tumor-infiltrating lymphocytes (TILs) exhibit significantly higher pCR rates and better survival outcomes (7, 8). A large pooled analysis further demonstrated that lymphocyte-rich triple-negative breast cancers (TNBCs) had markedly higher pCR rates than lymphocyte-poor tumors; even within HR-positive/HER2-negative subtypes, elevated TIL levels correlated with improved chemotherapy response (4). Similarly, both stromal and intratumoral TILs have been validated as independent predictors of pCR in TNBC (5). Collectively, these findings indicate that tumors with an immune activated microenvironment are more susceptible to chemotherapy. In addition to direct cytotoxic effects, chemotherapy can promote antitumor immunity by inducing immunogenic tumor cell death and enhancing immune activation (9). Anthracyclines such as doxorubicin have been shown to promote cytotoxic T-cell cross-priming while depleting immunosuppressive myeloid-derived suppressor cells (MDSCs), thereby enhancing antitumor immunity (10–12). Thus, chemotherapy can enhance antitumor immunity, and pre-existing immune activation may influence the likelihood of achieving pathologic complete response. This concept is supported by clinical trials showing improved pCR rates when immunotherapy is combined with neoadjuvant chemotherapy (13). These results underscore the central role of the immune system in mediating NAC efficacy and highlight immune-related molecular biomarkers as promising predictors of chemotherapy sensitivity and potential guides for individualized therapy.

Given the critical importance of the tumor immune microenvironment, growing efforts have focused on identifying immune-related biomarkers and signatures predictive of treatment response in HER2-negative breast cancer. On one hand, several studies have directly investigated immune predictors of NAC efficacy in HER2-negative tumors. For instance, Fang et al. analyzed gene expression profiles of HER2-negative breast cancer and intersected them with immune-related genes, identifying nine immune-associated genes—CXCL9, CXCL10, CXCL11, CXCL13, GZMB, IDO1, and LYZ—whose expression levels were significantly higher in pCR than in non-pCR samples; their predictive model achieved an AUC of 0.844 (14). On the other hand, most previous studies have focused on TILs, interferon-γ signaling, or antigen presentation pathways, yet their applicability to the HER2-negative population remains limited. For example, Matikas et al. evaluated pre-treatment immune infiltration and immune gene expression in HR-positive/HER2-negative tumors and found that higher TIL levels or upregulation of immune-related genes correlated with pCR, although the study was limited by small sample size and descriptive analysis without constructing or validating a robust predictive model specific to HER2-negative disease (15). Furthermore, studies combining immune checkpoint inhibitors with chemotherapy in high-risk breast cancer—including HER2-negative subtypes—suggest that immune activation may be a key determinant of therapeutic response, but research specifically focused on immune gene signatures predicting NAC response in HER2-negative patients remains scarce (16).

Therefore, reliable pretreatment biomarkers capable of predicting pathologic complete response remain an unmet clinical need in HER2 negative breast cancer. Currently used clinicopathological factors provide limited predictive accuracy and are insufficient to support individualized neoadjuvant treatment strategies (17). Given the central role of tumor immunity in chemotherapy sensitivity, immune related molecular signatures represent promising candidates for response prediction (16, 18). In the present study, we integrated large scale transcriptomic data from multiple independent cohorts of HER2 negative breast cancer patients treated with anthracycline and taxane based neoadjuvant chemotherapy. By combining immune gene profiling with machine learning approaches, we aimed to develop and validate an immune biomarker based predictive model for pathologic complete response and to explore the immunological features underlying treatment sensitivity.

## 2 Methods

### 2.1 Raw data

The gene expression data were obtained from the Gene Expression Omnibus (GEO) database. The dataset GSE194040, derived from the I-SPY2 Trial (“I-SPY2-990” subproject), includes pretreatment transcriptomic and proteomic/phosphoproteomic profiles from patients with early-stage invasive breast cancer undergoing neoadjuvant therapy. A total of 988 pretreatment breast cancer samples with comprehensive clinical annotations, including treatment arm, pathological complete response (pCR) status, molecular subtype, and MammaPrint (MP) classification, were available. To eliminate the confounding effects of anti-HER2 targeted therapy, we included only the 743 HER2-negative patients who received anthracycline- and taxane-based neoadjuvant chemotherapy, comprising 537 non-pCR and 206 pCR cases. For external validation, nine additional GEO datasets containing pretreatment transcriptomic profiles of HER2-negative breast cancer patients treated with anthracycline- and taxane-based neoadjuvant regimens (GSE20194, GSE20271, GSE22093, GSE23988, GSE25066, GSE32646, GSE34138, GSE41998, and GSE163882) were retrieved, encompassing a total of 1,642 patients (1,281 non-pCR and 361 pCR). To reduce platform related bias when integrating the external validation cohorts, expression matrices from the nine GEO validation datasets were merged using the intersection of shared genes. Batch effects across datasets were then corrected using the ComBat function in the sva package, with the dataset identifier specified as the batch variable. Principal component analysis was performed before and after batch correction to visually assess whether inter cohort technical variation was mitigated. The ANN model was trained on the independent training cohort, whereas the merged and batch corrected validation cohort served for external evaluation, aiming to assess generalizability under cross platform conditions after mitigation of batch effects. Moreover, a comprehensive list of immune-related genes was downloaded from the ImmPort database (https://www.immport.org) for subsequent analyses.

### 2.2 Differential expression analysis

To identify the transcriptomic differences associated with chemotherapy response across hormone receptor (HR) subtypes, the 743 HER2-negative breast cancer patients from the GSE194040 dataset were stratified into 364 HR-negative and 379 HR-positive cases. Within each HR subgroup, differential expression analysis between pCR and non-pCR samples was performed using the limma R package. Gene expression matrices were normalized with the normalizeBetweenArrays function, and differentially expressed genes (DEGs) were identified based on |log_2_FC| > 1 and adjusted p < 0.05 (Benjamini–Hochberg correction) (19). The thresholds of |log_2_ fold change| greater than 1 and adjusted p value less than 0.05 were selected to ensure that identified differentially expressed genes represented biologically meaningful expression changes while maintaining adequate statistical stringency. Applying the same criteria across hormone receptor positive and hormone receptor negative subgroups allowed for consistent comparison of transcriptional differences associated with treatment response, despite intrinsic biological heterogeneity between subtypes. Heatmaps and volcano plots were generated using pheatmap and ggplot2 to visualize the expression patterns and statistical significance of DEGs (20). Subsequently, the DEGs from the HR-positive and HR-negative cohorts were compared, and genes that were significantly dysregulated in both groups with concordant regulation direction were extracted as overlapping DEGs for downstream analyses. All computations and visualizations were conducted in R (version 4.5.0).

### 2.3 Weighted gene co-expression network analysis

To further identify gene co-expression modules associated with chemotherapy response, we performed a weighted gene co-expression network analysis (WGCNA) based on the normalized transcriptomic profiles of 743 HER2-negative breast cancer patients (pCR vs. non-pCR) from the GSE194040 dataset (21). Genes with low variance (standard deviation < 0.3) were excluded to improve network robustness. Outlier samples were detected and removed through hierarchical clustering. The optimal soft-thresholding power (β) was determined using the pickSoftThreshold function to ensure a scale-free network topology (R² > 0.9). An adjacency matrix was constructed and transformed into a topological overlap matrix (TOM) to quantify gene interconnectedness, followed by hierarchical clustering to identify gene modules using the dynamicTreeCut algorithm (minimum module size = 50). Highly similar modules were merged at a cut height of 0.3 to obtain the final module structure. The relationships between module eigengenes and clinical traits (pCR and non-pCR status) were calculated using Pearson correlation, and a module–trait heatmap was generated to visualize the strength and significance of associations. Module significance and gene significance were computed to identify modules most strongly correlated with therapeutic response (22). Modules were prioritized for downstream analyses based on the strength and direction of their correlation with pCR status, statistical significance, and overall module significance, with preference given to modules showing the most robust and biologically plausible associations with treatment response.

### 2.4 Identification of candidate diagnostic genes using machine learning algorithms

To obtain robust candidate genes associated with neoadjuvant chemotherapy response, the genes from the module most significantly correlated with pCR in the WGCNA (23), the directionally concordant DEGs shared by HR-positive and HR-negative subgroups, and the immune-related genes from the ImmPort database were intersected to generate a key gene set for feature selection. Three complementary machine learning algorithms were then applied. First, least absolute shrinkage and selection operator (LASSO) regression was performed using the glmnet package in R (24), with tenfold cross-validation to identify the optimal penalty parameter (λ) that minimized deviance, and genes with nonzero coefficients at λ_min were retained. Second, a random forest (RF) classifier was constructed using the randomForest package with 500 decision trees to evaluate the importance of each gene based on the mean decrease in Gini index, and genes with importance scores above the optimal cutoff were selected. Third, a support vector machine–recursive feature elimination (SVM-RFE) approach was implemented via the e1071 package and an in-house script to iteratively eliminate less informative features and determine the optimal subset of genes with the lowest cross-validation error. Genes consistently identified by LASSO, RF, and SVM-RFE were considered as key feature genes for subsequent modeling and validation.

### 2.5 Artificial neural network model construction and validation

To develop and validate a predictive model for chemotherapy response, the intersecting genes jointly identified by the LASSO, RF, and SVM-RFE algorithms were used to construct an artificial neural network (ANN). The normalized expression profiles of 743 HER2-negative breast cancer patients from the GSE194040 dataset (training cohort) served as the training data, while an independent validation cohort comprising 1,642 HER2-negative breast cancer patients from nine GEO datasets (GSE20194, GSE20271, GSE22093, GSE23988, GSE25066, GSE32646, GSE34138, GSE41998, and GSE163882) was used for external validation. A feed-forward ANN model was implemented using the neuralnet package in R, with two output nodes (pCR and non-pCR) and a single hidden layer containing three neurons. Model training was performed with a maximum of 1,000,000 iterations to ensure convergence. The network structure and connection weights were visualized using the NeuralNetTools package. The trained model was then applied to the external validation dataset to predict pCR probabilities, and model performance was assessed using receiver operating characteristic (ROC) analysis via the pROC package. The area under the curve (AUC) and corresponding 95% confidence interval (calculated using the bootstrap method) were used to evaluate the discriminative ability and generalizability of the ANN model in predicting pCR among HER2-negative breast cancer patients (25).

The architecture of the artificial neural network was intentionally kept simple to reduce the risk of overfitting and to improve model interpretability. The number of hidden neurons was selected empirically through preliminary testing, in which multiple network configurations with different hidden layer sizes were evaluated for stability and predictive performance. A single hidden layer with three neurons provided a favorable balance between model complexity and generalization ability and did not yield substantial performance improvement when additional neurons were introduced. The maximum number of training iterations was set to 1,000,000 to ensure convergence of the network weights during optimization. Model training typically converged well before reaching the maximum iteration limit, which was used as an upper bound rather than a fixed stopping point. This strategy ensured stable model training without forcing unnecessary complexity. These hyperparameter choices were guided by a combination of empirical optimization within the training cohort and prior experience from published studies applying neural network models to transcriptomic data with limited feature dimensions.

### 2.6 Key gene expression and immune infiltration analyses

To investigate the biological relevance of the identified feature genes, we first validated their expression differences between pCR and non-pCR groups in both the training and external validation cohorts. Normalized expression matrices were subset to the intersected feature genes, and two-sided Wilcoxon rank-sum tests were applied to compare gene expression levels, with results visualized as boxplots using the ggpubr package. Subsequently, immune infiltration analysis was performed only in the training cohort. The proportions of 22 immune cell types were estimated using the CIBERSORT algorithm (1,000 permutations) based on the LM22 leukocyte gene signature matrix (26). Differences in immune-cell fractions between pCR and non-pCR samples were evaluated using Wilcoxon tests, and boxplots were generated to display significantly altered immune populations. Finally, Spearman correlation analysis was conducted to examine associations between model gene expression and immune-cell infiltration levels, and the correlation network was visualized using the linkET package. To account for multiple hypothesis testing when comparing the relative proportions of 22 immune cell types, p values were further adjusted using the Benjamini–Hochberg false discovery rate method. Both nominal and FDR adjusted p values were considered when interpreting immune infiltration differences between groups.

### 2.7 Gene set enrichment analysis based on key genes

GSEA was performed in the training cohort to interrogate pathways associated with the five key genes. For each gene, samples were dichotomized into high- and low-expression groups by the cohort median; because all five genes were overexpressed in pCR, we focused on the high-expression groups. A ranked gene list was generated by the difference in mean expression between high and low groups, and enrichment was tested with clusterProfiler (using MSigDB KEGG collection c2.cp.kegg.Hs.symbols.gmt, https://www.gsea-msigdb.org/gsea/msigdb/collections.jsp) (27). Results were summarized by normalized enrichment score (NES) and significance (nominal p < 0.05; adjusted q values were reported when available), and the top enriched pathways (up to five per gene) were visualized with enrichplot (gseaplot2). Analyses used R with limma for preprocessing and org.Hs.eg.db for gene annotation. For enrichment analyses, adjusted p values calculated using the Benjamini–Hochberg method were used to define statistical significance unless otherwise specified. Nominal p values were reported only for descriptive purposes and were explicitly labeled as such in the corresponding Results.

### 2.8 Drug enrichment, ceRNA, and transcription factor network analyses

To further explore the regulatory mechanisms and therapeutic relevance of the key genes, drug enrichment, ceRNA, and transcription factor (TF) network analyses were performed. Drug enrichment was conducted using the DSigDB database (https://dsigdb.tanlab.org/) with the clusterProfiler package, applying the enricher function based on the DSigDB_All_detailed dataset. Significantly enriched drugs were defined as those with p < 0.05 and adjusted p < 0.05, and visualized using enrichplot and ggplot2. For post-transcriptional regulation, a ceRNA network was constructed by integrating miRNA–mRNA interactions predicted by miRanda, miRDB, TargetScan, and miRWalk, retaining miRNAs identified in at least four databases. Corresponding lncRNA–miRNA interactions were obtained from spongeScan, and the merged network was visualized in Cytoscape (v3.10.1). Upstream transcriptional regulation was investigated using the TRRUST v2 database (https://www.grnpedia.org/trrust/), where curated TF–target relationships were retrieved, and upstream TFs regulating the key mRNAs were identified to construct the TF–gene regulatory network for visualization and interpretation.

### 2.9 qRT-PCR validation of key genes

To experimentally validate the expression of key predictive genes, pretreatment tumor tissue samples were collected from 60 HER2-negative breast cancer patients, including 30 patients who achieved a pathological complete response (pCR) and 30 patients who did not achieve pCR (non-pCR) following anthracycline- and taxane-based neoadjuvant chemotherapy (28). Total RNA was extracted from tumor tissues and reverse-transcribed into complementary DNA. Quantitative real-time PCR (qRT-PCR) was performed to measure the mRNA expression levels of five immune-related genes (CCL2, CXCL10, CXCL13, HLA-E, and IGKV1D-8) using gene-specific primers. The primers used were as follows:

CCL2, forward 5′-CATCTCCTACACCCCACGAAG-3′ and reverse 5′-GGGTTGGCACAGAAACGTC-3′;

CXCL10, forward 5′-GCTCTAGAATTGCTGCCTTATCTTTCT-3′ and reverse 5′-CTCAACACGTGGGCAGGATTA-3′;

CXCL13, forward 5′-CTCAACGTCCCATCTACTTGC-3′ and reverse 5′-TCTTCAGGGTGTGAGCTTTCC-3′;

HLA-E, forward 5′-CCTGGAGGAGATACGGGAGAC-3′ and reverse 5′-GGACAGCCCTCCATGGT-3′;

IGKV1D-8, forward 5′-TCCTGGGGCTCTCATGCT-3′ and reverse 5′-GGATGGTGGGAAGATGGA-3′.

ACTB (β-actin) was amplified with forward primer 5′-AAGGAGCCCCACGAGAAAAAT-3′ and reverse primer 5′-ACCGAACTTGCATTGATTCCAG-3′.

All reactions were run in triplicate. Expression levels were normalized to ACTB and calculated using the 2^−ΔΔCt^ method. Differences in relative mRNA expression between the pCR (n = 30) and non-pCR (n = 30) groups were compared with two-sided Wilcoxon rank-sum tests.

### 2.10 Statistical analysis

All statistical analyses were conducted using R software (version 4.5.0). Differences in gene expression and immune-cell infiltration between groups were evaluated using two-sided Wilcoxon rank-sum tests. The relationships between module eigengenes and clinical traits in the WGCNA were assessed using Pearson correlation analysis. Gene set enrichment analysis (GSEA) and drug enrichment analysis were performed using the clusterProfiler package, with significance defined as nominal *p* < 0.05 and adjusted *p* < 0.05. Correlations between key gene expression levels and immune-cell fractions were examined using Spearman’s rank correlation. All tests were two-tailed, and a *p* < 0.05 was considered statistically significant.

## 3 Results

### 3.1 Identification of DEGs between HR subtypes

**Figure 1:**
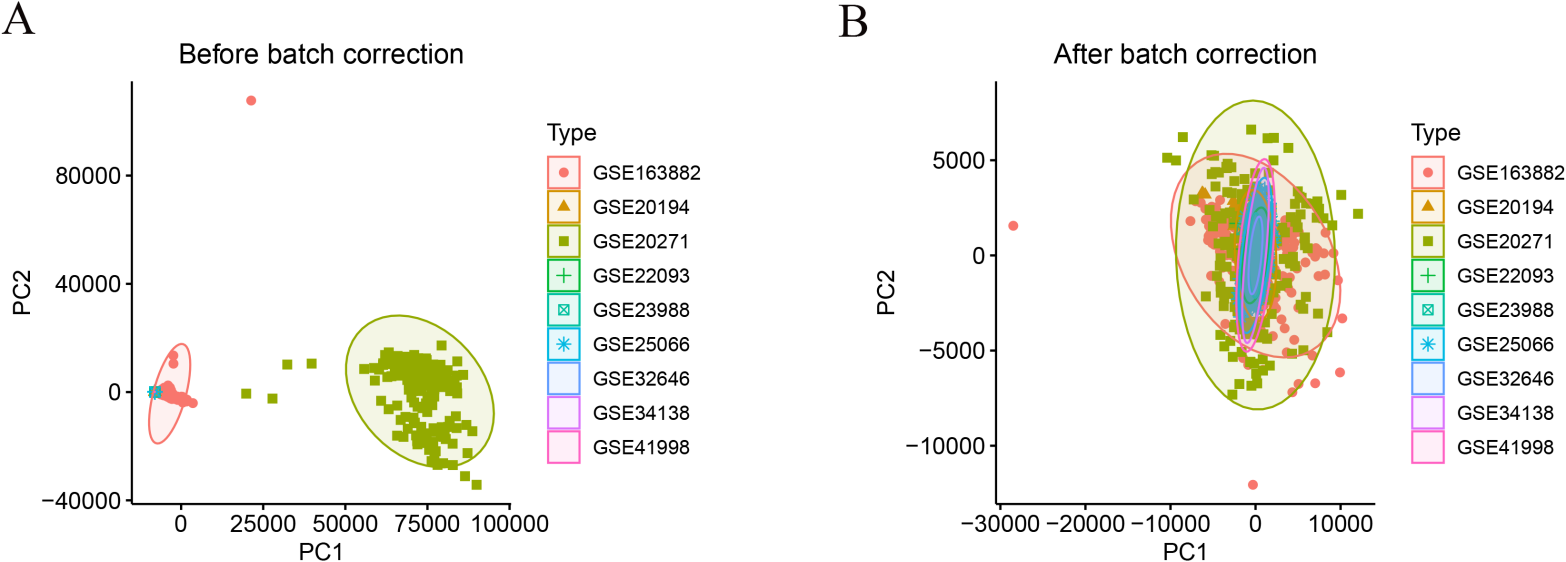
PCA diagnostics before and after batch correction in the merged external validation cohorts.

**Figure 2:**
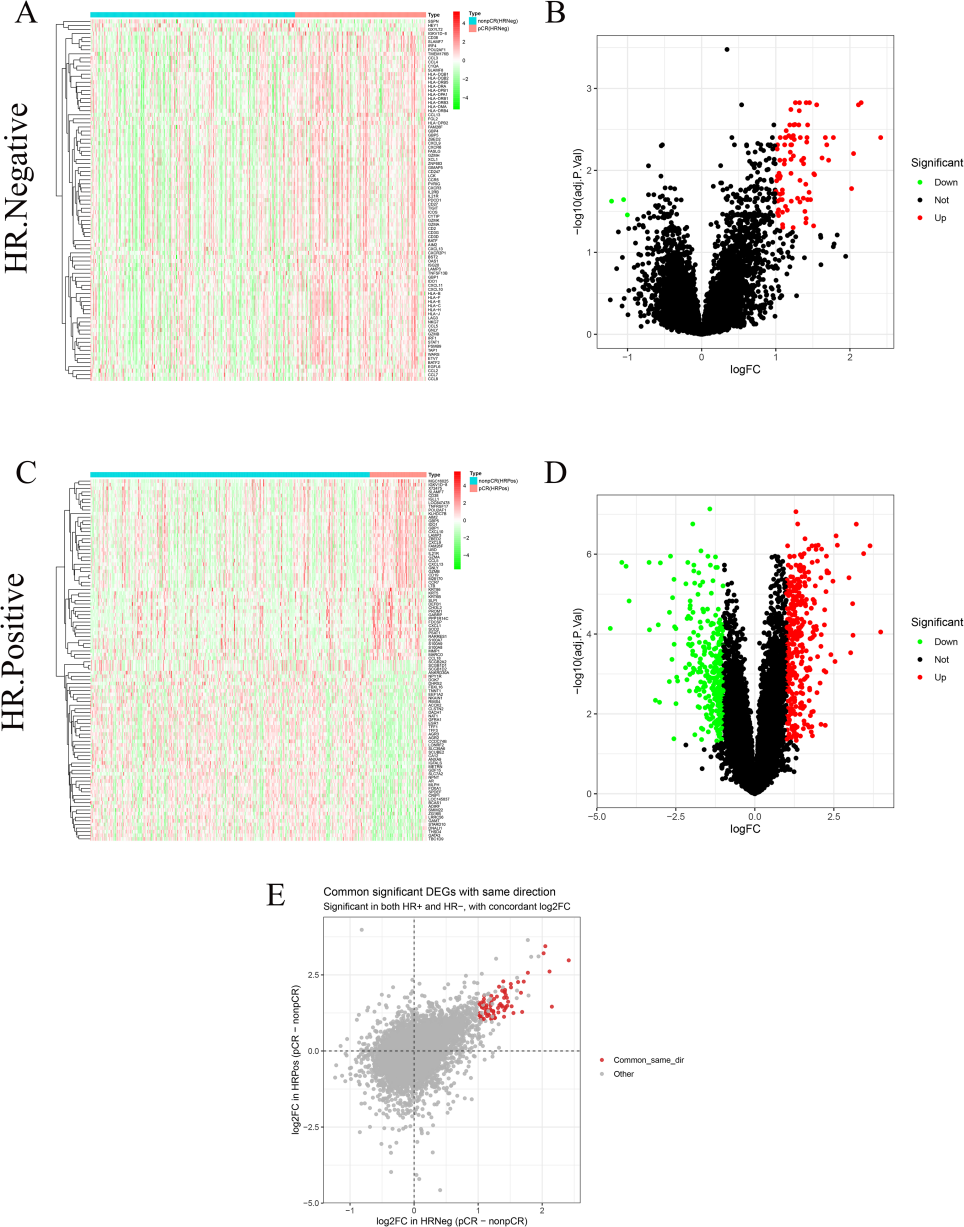
Identification of DEGs between HR-negative and HR-positive subtypes. **(A, B)** Heatmap and volcano plot showing DEGs between pCR and non-pCR samples in the HR-negative subgroup. **(C, D)** Heatmap and volcano plot showing DEGs between pCR and non-pCR samples in the HR-positive subgroup. **(E)** Scatter plot showing overlapping DEGs with consistent regulation between the two subtypes.

To evaluate and mitigate potential batch effects among the nine external GEO validation cohorts, we merged these datasets based on the common gene set and applied ComBat batch correction using the cohort identifier as the batch factor. As shown in **Figures 1A, B**, samples exhibited clear cohort driven separation before correction, whereas the inter cohort clustering pattern was substantially attenuated after correction, indicating reduced platform related bias. To explore transcriptional differences associated with neoadjuvant chemotherapy response, differential expression analysis was performed separately in HR-negative and HR-positive HER2-negative breast cancer subgroups from the GSE194040 dataset. In HR- positive tumors, a total of 669 DEGs were identified (|log_2_FC| > 1, adjusted p < 0.05), including 362 upregulated and 307 downregulated genes, clearly separating pCR from non-pCR samples (**Figures 2A, B**). Similarly, in HR-negative tumors, 89 DEGs were identified (86 upregulated and 3 downregulated), demonstrating distinct expression patterns between response groups (**Figures 2C, D**). Comparison of the two DEG sets revealed 77 overlapping genes with concordant regulation direction (**Figure 2E**), indicating a shared transcriptional signature related to chemotherapy sensitivity across HR subtypes. The difference in the number of differentially expressed genes between hormone receptor positive and hormone receptor negative tumors likely reflects inherent biological differences in transcriptional variability and immune activation between these subtypes rather than analytical bias introduced by the applied thresholds.

### 3.2 Weighted gene co-expression network analysis

**Figure 3:**
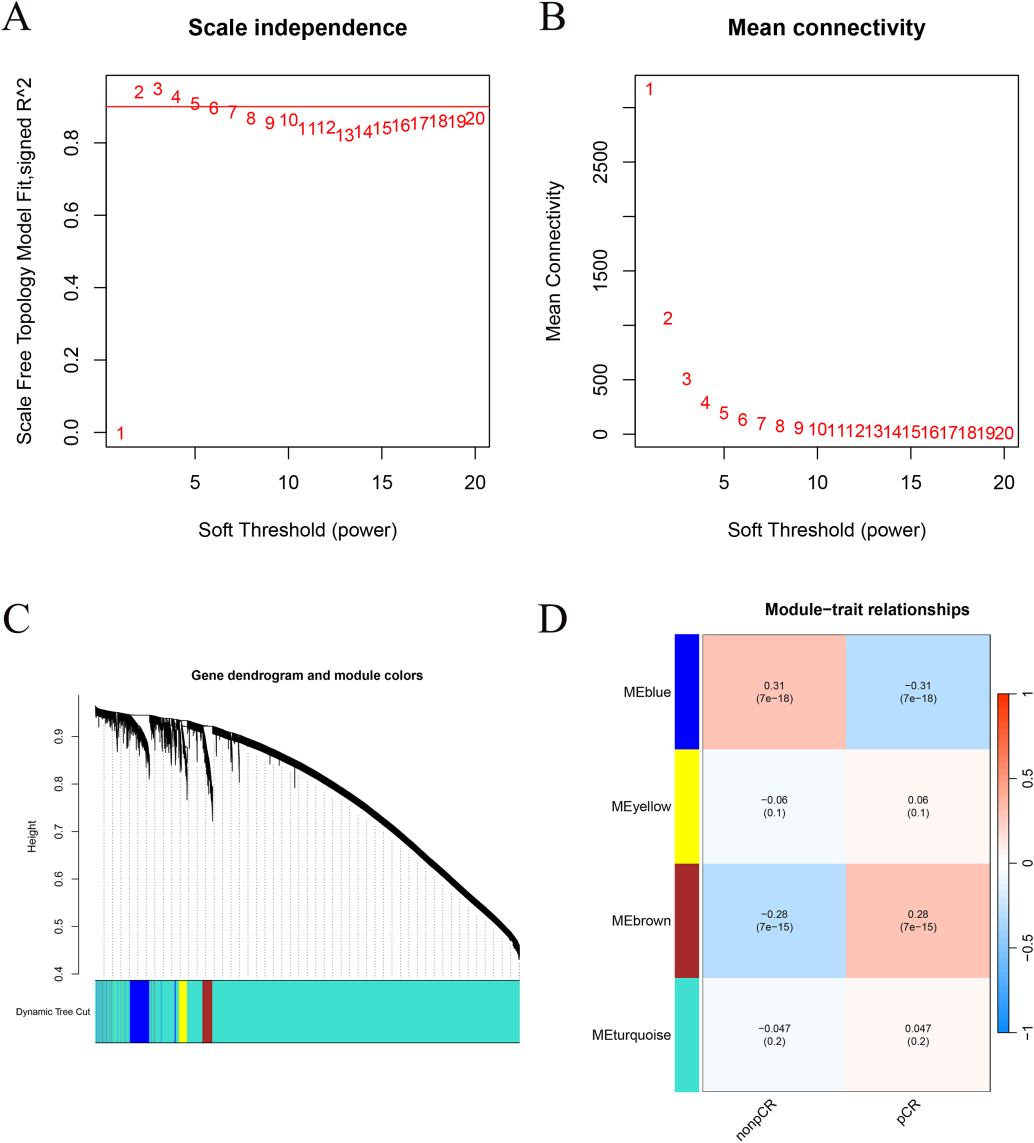
Weighted gene co-expression network analysis (WGCNA). **(A)** Scale-free topology fit index (R²) versus soft-thresholding power. **(B)** Mean connectivity at each power. **(C)** Dendrogram of genes clustered into distinct modules. **(D)** Heatmap of module–trait correlations showing the blue module most correlated with pCR status.

WGCNA was conducted using expression data from 743 HER2-negative patients to identify gene co-expression modules associated with pCR. The scale-free topology criterion (R² > 0.9) was achieved at β = 4 (**Figures 3A, B**). Hierarchical clustering identified four major co-expression modules (blue, brown, yellow, and turquoise; **Figure 3C**). Among them, the blue module showed the strongest positive correlation with pCR (r = 0.31, p < 0.01) and negative correlation with non-pCR (r = −0.31; **Figure 2D**). Among all identified modules, the blue module showed the strongest positive correlation with pathologic complete response, with a correlation coefficient of 0.31 and a statistically significant p value. This module contained a total of 1004 genes and exhibited a higher module significance and gene significance for pCR compared with other modules, supporting its prioritization for downstream analyses.

### 3.3 Integration of WGCNA, immune genes, and machine learning feature selection

**Figure 4:**
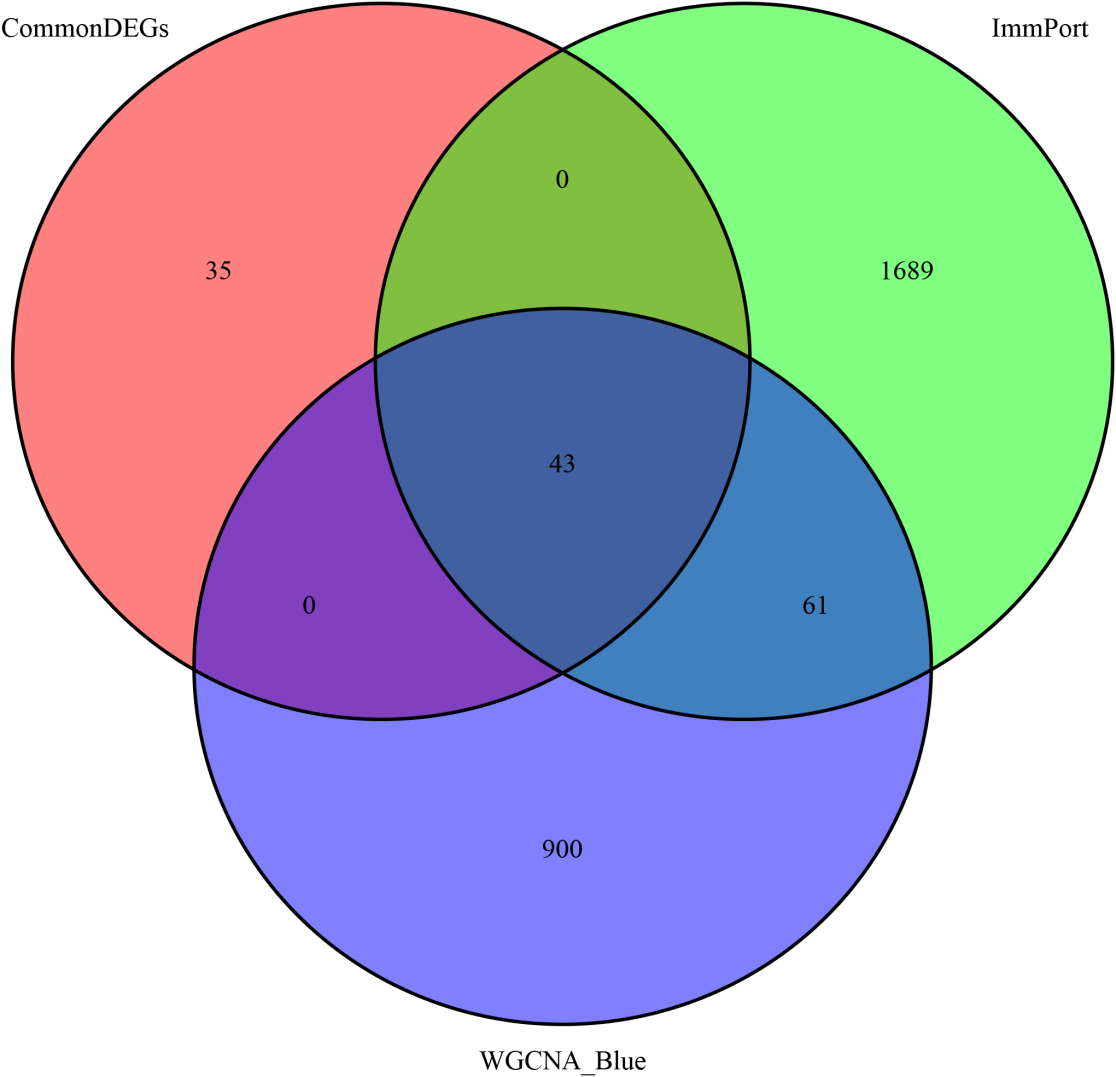
Integration of immune-related, DEG, and WGCNA genes. Venn diagram showing 43 overlapping genes among ImmPort immune genes, common DEGs, and WGCNA blue module genes.

**Figure 5:**
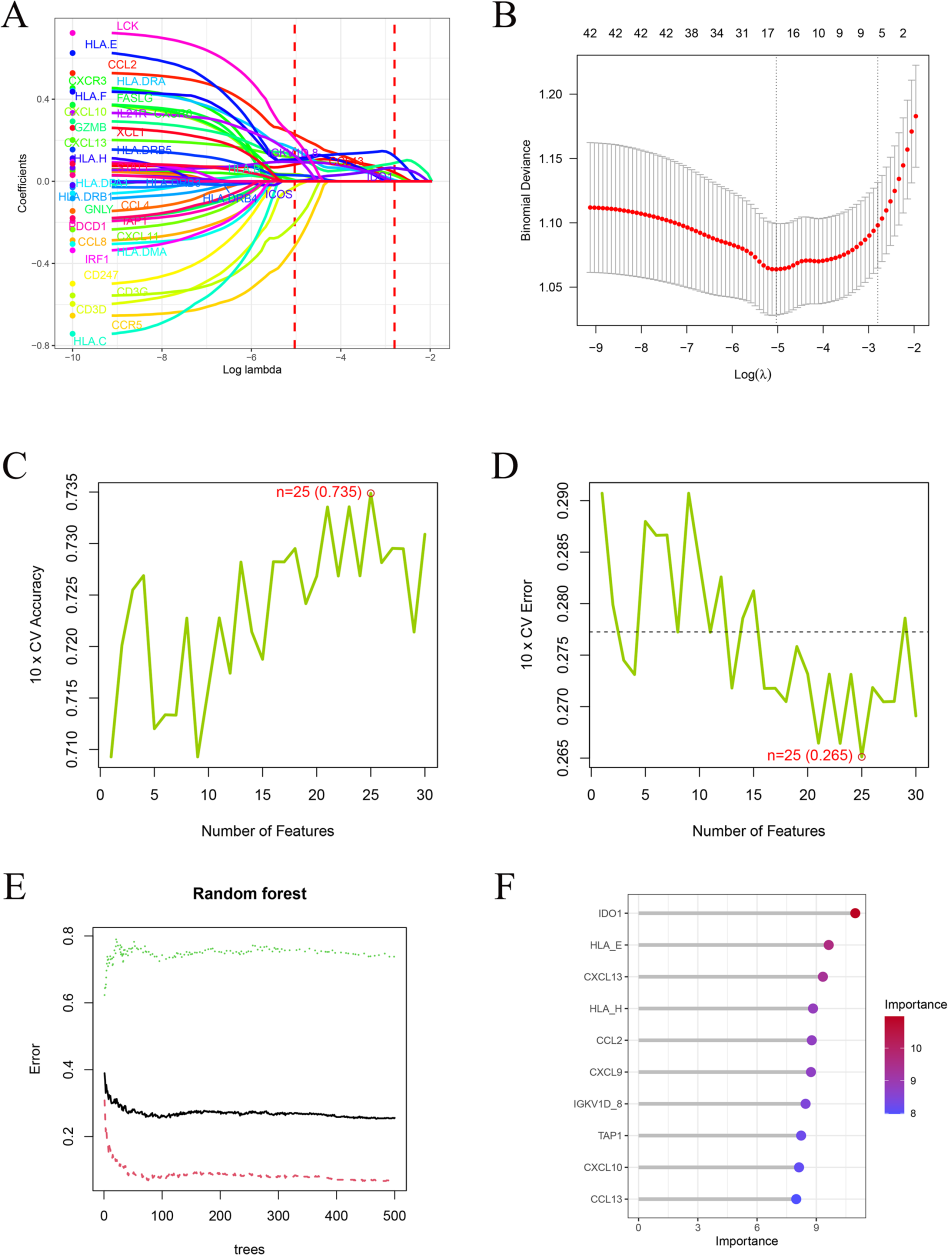
Machine learning–based feature gene selection. **(A, B)** LASSO regression analysis identifying genes with nonzero coefficients at the optimal λ. **(C, D)** SVM-RFE showing optimal feature subset with maximum accuracy and minimum error. **(E, F)** Random Forest error curves and variable importance ranking.

To identify robust immune biomarkers associated with chemotherapy response, genes from the WGCNA blue module, the ImmPort immune gene list, and the common DEGs with consistent regulation direction between HR subtypes were intersected, yielding 43 overlapping genes (**Figure 4**). These genes were subsequently subjected to multi-algorithmic feature selection. LASSO regression identified 17 genes with nonzero coefficients at the optimal λ (**Figures 5A, B**), while SVM-RFE achieved the highest classification accuracy (0.735) and lowest error (0.265) with 25 features (**Figures 5C, D**). Random forest analysis ranked gene importance and selected the top ten feature genes (**Figures 5E, F**), which were subsequently used for model construction and validation.

### 3.4 Construction and validation of the artificial neural network model

**Figure 6:**
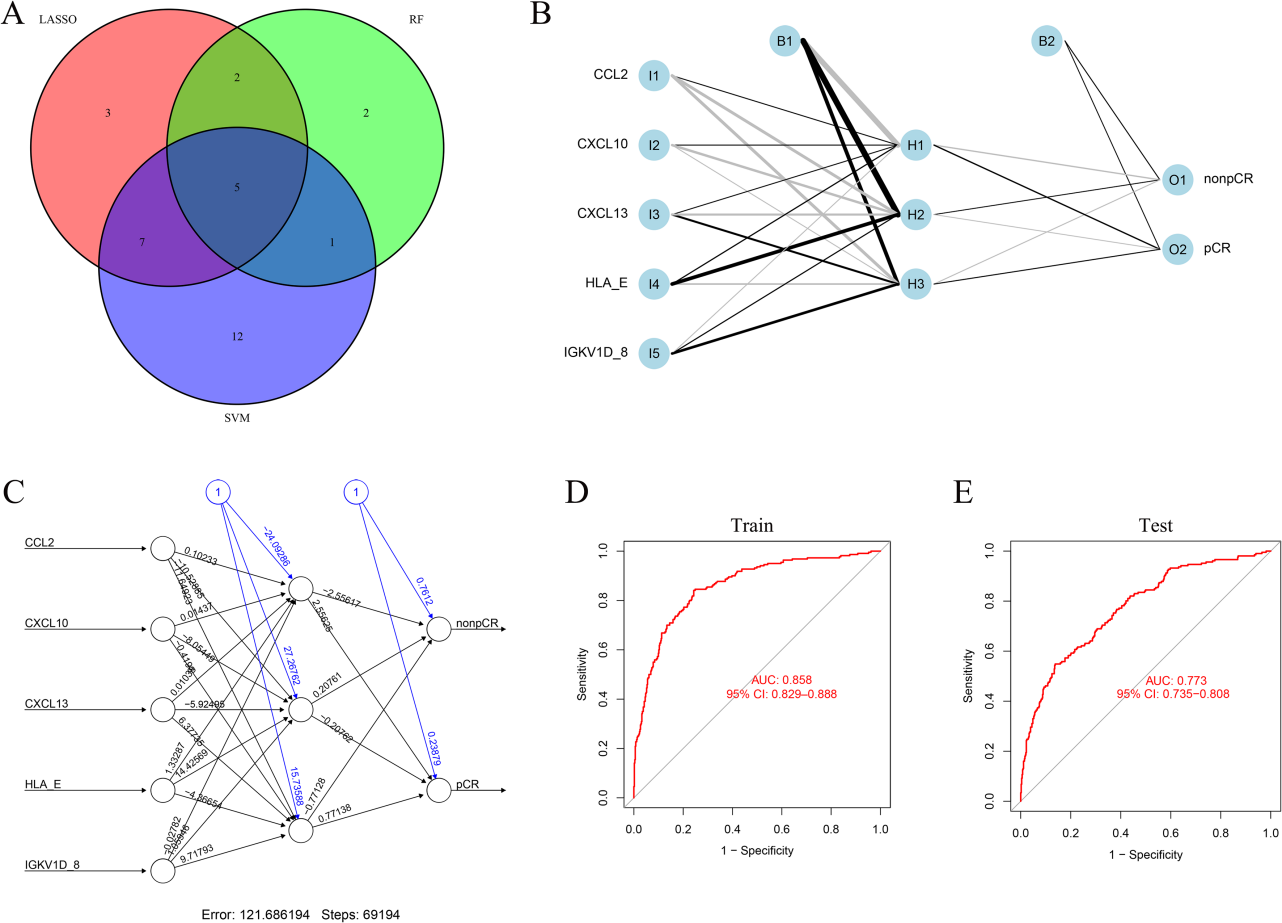
ANN model construction and validation. **(A)** Venn diagram showing intersection of LASSO, RF, and SVM-RFE results. **(B)** ANN architecture with five input genes, one hidden layer, and two output nodes. **(C)** Visualization of network weights. **(D, E)** ROC curves showing model performance in training and validation cohorts.

The intersection of LASSO, RF, and SVM-RFE analyses identified five shared key genes—CCL2, CXCL10, CXCL13, HLA-E, and IGKV1D-8—which were subsequently used to construct an artificial neural network (ANN) model for predicting chemotherapy response (**Figure 6A**). The ANN was developed using the GSE194040 cohort (n = 743) as the training set and externally validated in nine independent GEO datasets (n = 1,642). The model comprised five input nodes corresponding to the key genes, a single hidden layer with three neurons, and two output nodes representing pCR and non-pCR outcomes (**Figures 6B, C**). The ANN exhibited strong discriminative performance, achieving an AUC of 0.858 (95% CI: 0.829-0.888) in the training cohort and 0.773 (95% CI: 0.735–0.808) in the validation cohort (**Figures 6D, E**), indicating its robust and generalizable predictive capacity across independent datasets.

### 3.5 Expression validation and immune infiltration analysis

**Figure 7:**
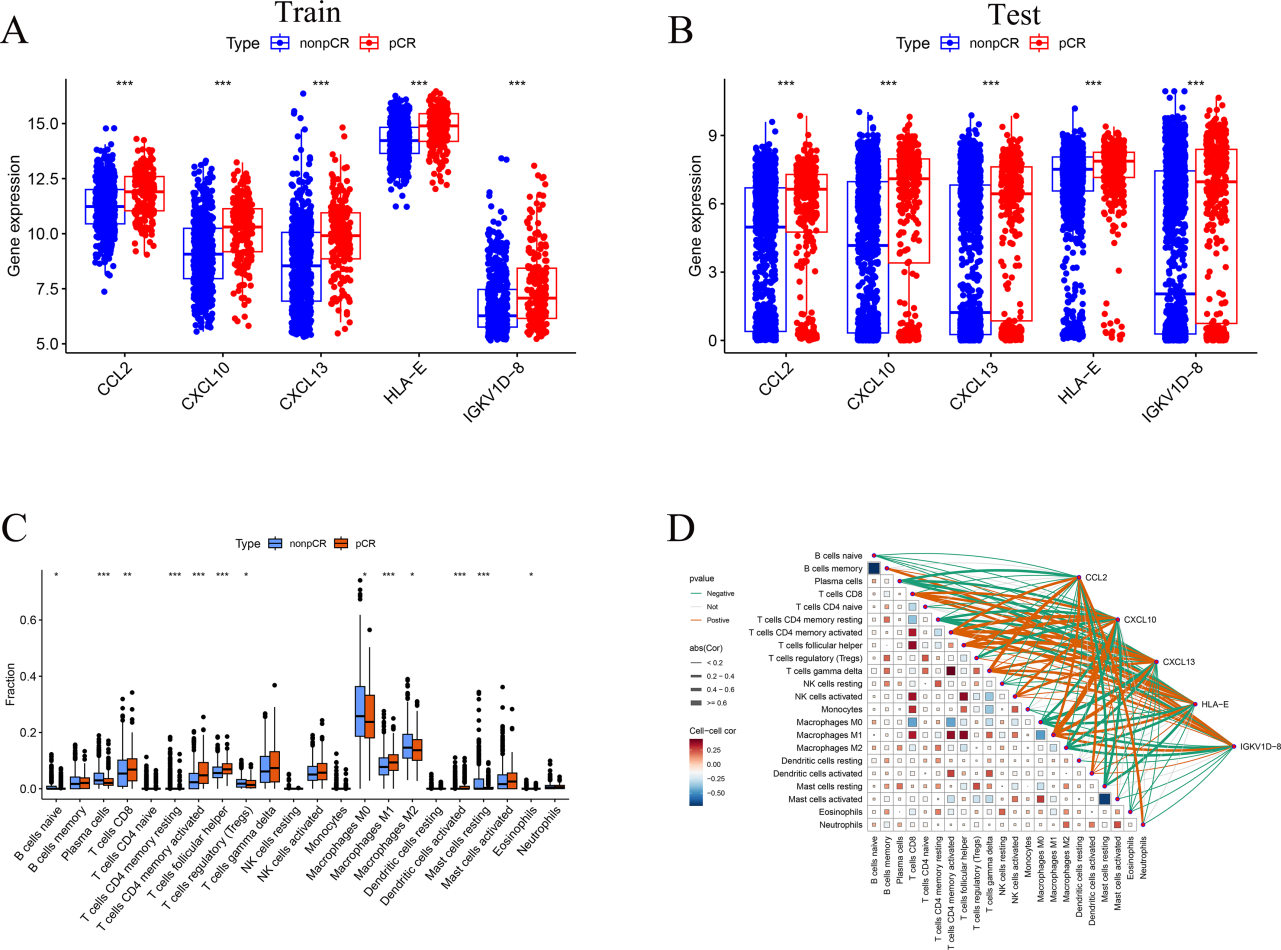
Expression and immune infiltration analyses. **(A, B)** Expression of five key genes between pCR and non-pCR groups in training and validation cohorts. **(C)** Boxplots show the relative fractions of 22 immune cell types estimated by CIBERSORT. Differences between groups were assessed using the Wilcoxon rank sum test. Statistical significance is indicated as follows: *p < 0.05, **p < 0.01, ***p < 0.001. P values shown are nominal unless otherwise specified, and results should be interpreted in the context of multiple testing. **(D)** Correlation network between key genes and immune cell populations. Gene nodes and immune cell nodes are distinguished by distinct shapes and color schemes. Edge color indicates the direction of correlation, with one color representing positive correlations and another representing negative correlations. Edge thickness reflects the absolute value of the correlation coefficient. Only correlations meeting the predefined significance threshold are displayed. A detailed legend is provided to explain all visual encodings.

In both the training and external validation cohorts, all five key genes were significantly upregulated in the pCR group compared with the non-pCR group (**Figures 7A, B**). CIBERSORT-based immune infiltration analysis revealed distinct immune cell distributions between response groups (**Figure 7C**). The pCR group showed higher proportions of T cells CD8, T cells CD4 memory activated, T cells follicular helper, M1 macrophages, and activated dendritic cells, reflecting an inflammatory and immune-stimulating microenvironment. In contrast, non-pCR tumors exhibited increased infiltration of naïve B cells, Plasma cell, T cells CD4 memory resting, T cells regulatory (Tregs), M0 and M2 macrophages, and Mast cells resting, suggesting an immunosuppressive state. Correlation network analysis (**Figure 7D**) further revealed that CCL2, CXCL10, CXCL13, HLA-E, and IGKV1D-8 were positively correlated with multiple effector and antigen-presenting immune subsets, including T cells CD8, T cells CD4 memory activated, T cells follicular helper, Macrophages M1, and Dendritic cells activated, indicating their close association with immune activation and cytotoxic responses. Conversely, these genes were negatively correlated with B cells naïve, Plasma cells, T cells CD4 memory resting, T cells regulatory (Tregs), Macrophages M0, Macrophages M2, and Mast cells resting, which are characteristic of an immunosuppressive microenvironment. Collectively, these findings suggest that high expression of the five key genes is linked to a pro-inflammatory, antitumor immune milieu favorable for chemotherapy responsiveness.

### 3.6 Gene set enrichment analysis

**Figure 8:**
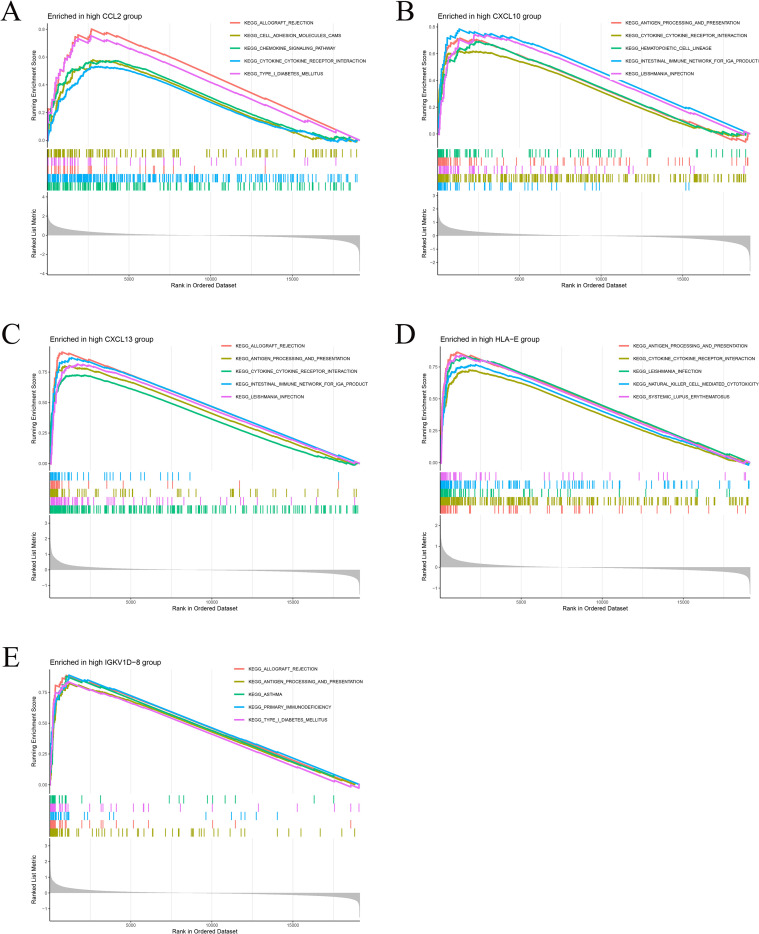
Gene set enrichment analysis (GSEA). **(A–E)** GSEA plots showing significantly enriched KEGG pathways in the high-expression groups of the five key genes. **(A)** High CCL2 group: allograft rejection, chemokine signaling, and cytokine–cytokine receptor interaction. **(B)** High CXCL10 group: antigen processing and presentation, cytokine–cytokine receptor interaction, and hematopoietic cell lineage. **(C)** High CXCL13 group: allograft rejection, antigen processing and presentation, and intestinal immune network for IgA production. **(D)** High HLA-E group: antigen processing and presentation, cytokine–cytokine receptor interaction, and natural killer cell–mediated cytotoxicity. **(E)** High IGKV1D-8 group: allograft rejection, antigen processing and presentation, and primary immunodeficiency.

GSEA revealed that the high‐expression groups of the five key genes were significantly enriched in immune‐related pathways (**Figures 8A–E**). Enriched pathways were considered statistically significant based on adjusted p values, whereas pathways reported using nominal p values are explicitly indicated and interpreted with caution. Specifically, the high CCL2 group was enriched in allograft rejection, cell adhesion molecules (CAMs), chemokine signaling pathway, cytokine–cytokine receptor interaction, and type I diabetes mellitus. The high CXCL10 group showed enrichment in antigen processing and presentation, cytokine–cytokine receptor interaction, hematopoietic cell lineage, intestinal immune network for IgA production, and Leishmania infection. The high CXCL13 group was enriched in allograft rejection, antigen processing and presentation, cytokine–cytokine receptor interaction, intestinal immune network for IgA production, and Leishmania infection. The high HLA-E group exhibited enrichment in antigen processing and presentation, cytokine–cytokine receptor interaction, Leishmania infection, natural killer cell–mediated cytotoxicity, and systemic lupus erythematosus. Finally, the high IGKV1D-8 group was enriched in allograft rejection, antigen processing and presentation, asthma, primary immunodeficiency, and type I diabetes mellitus.

### 3.7 Regulatory network and drug enrichment analysis

**Figure 9:**
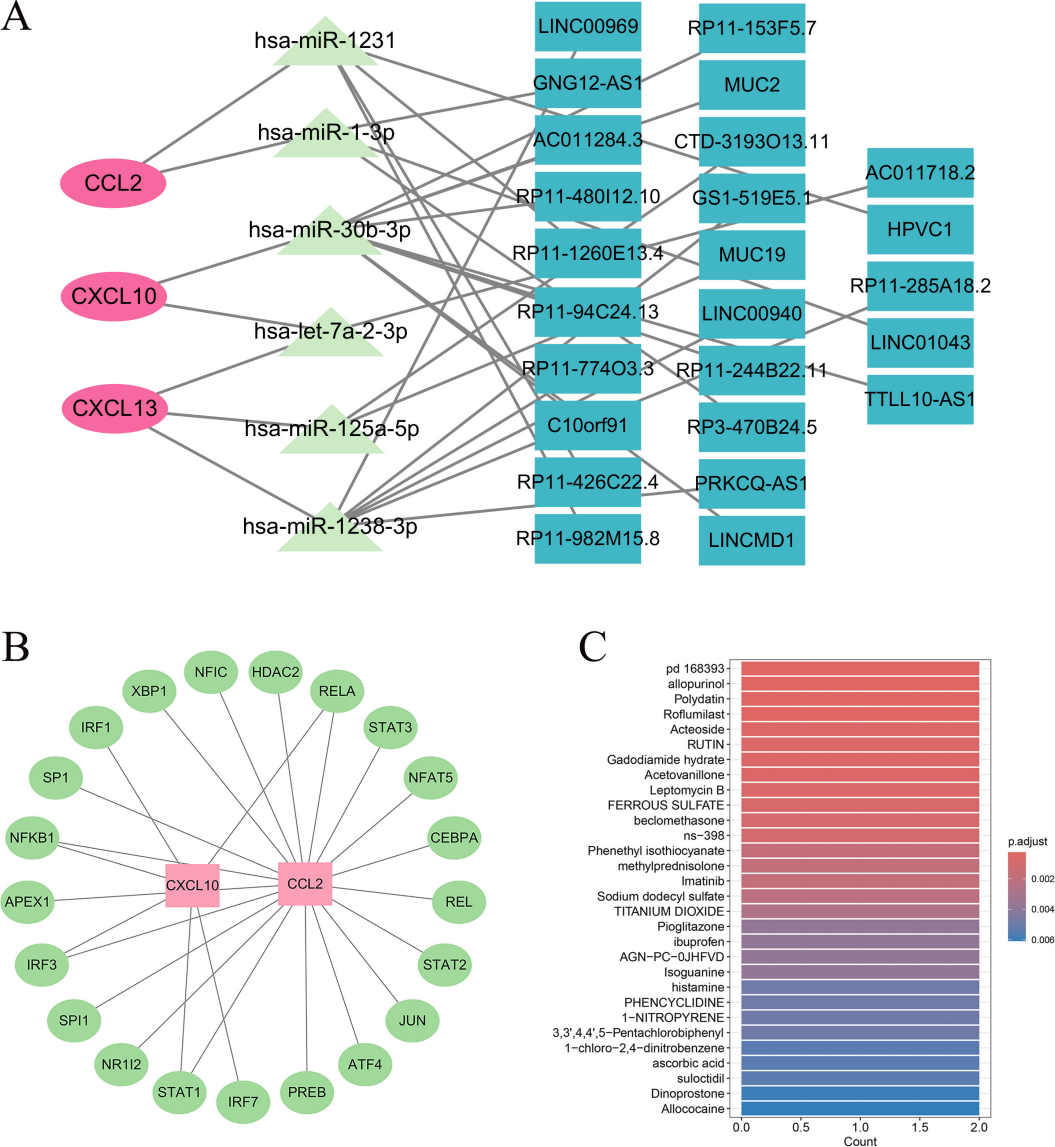
Regulatory and drug enrichment analyses. **(A)** ceRNA network showing interactions among mRNAs, miRNAs, and lncRNAs. **(B)** Transcription factor–gene regulatory network identifying major upstream TFs. **(C)** Bubble plot showing top enriched drugs associated with key genes.

A comprehensive ceRNA network was constructed to explore the post-transcriptional regulatory relationships of the key genes (**Figure 9A**). The network included 3 mRNAs (CCL2, CXCL10, CXCL13), 26 miRNAs, and 42 lncRNAs, forming a multilayered regulatory architecture. Among them, CCL2 was potentially regulated by multiple miRNAs such as hsa-miR-125a-5p, hsa-miR-30b-3p, and hsa-miR-195-5p, which were further modulated by lncRNAs including LINC00969, AC011284.3, and HCP5. CXCL10 was predicted to be targeted by hsa-miR-29b-3p, hsa-miR-34a-5p, and hsa-miR-340-5p, while CXCL13 was associated with hsa-miR-101-3p, hsa-miR-155-5p, and hsa-miR-450a-5p, all of which were interconnected with lncRNAs such as MIR155HG, LINC00511, and AC006449.2. These results delineate a complex ceRNA regulatory landscape potentially influencing gene expression and immune activity. Transcriptional regulation analysis based on the TRRUST v2 database identified several upstream transcription factors (TFs) strongly associated with the key genes (**Figure 9B**). CCL2 and CXCL10 were mainly regulated by NF-κB1, RELA, STAT1, STAT3, and IRF1, whereas CXCL13 was primarily controlled by IRF4 and SPI1. These TFs are well known for their roles in inflammatory and interferon-mediated signaling, suggesting transcriptional activation of these chemokine genes under immune-stimulating conditions. The drug enrichment analysis performed using the DSigDB database revealed a series of compounds potentially targeting the key genes (**Figure 9C**). The top significantly enriched molecules included pioglitazone, ibuprofen, roflumilast, rutin, methylprednisolone, beclomethasone, allopurinol, and acteoside. Several of these agents, such as pioglitazone (a PPAR-γ agonist) and roflumilast (a phosphodiesterase-4 inhibitor), have recognized immunomodulatory or anti-inflammatory effects, whereas ibuprofen and rutin were associated with cytokine pathway regulation. The enrichment of these compounds suggests that modulation of inflammatory and chemokine signaling might enhance chemotherapy sensitivity in HER2-negative breast cancer.

### 3.8 qRT-PCR validation of key immune genes in HER2 negative tumor tissues

**Figure 10:**
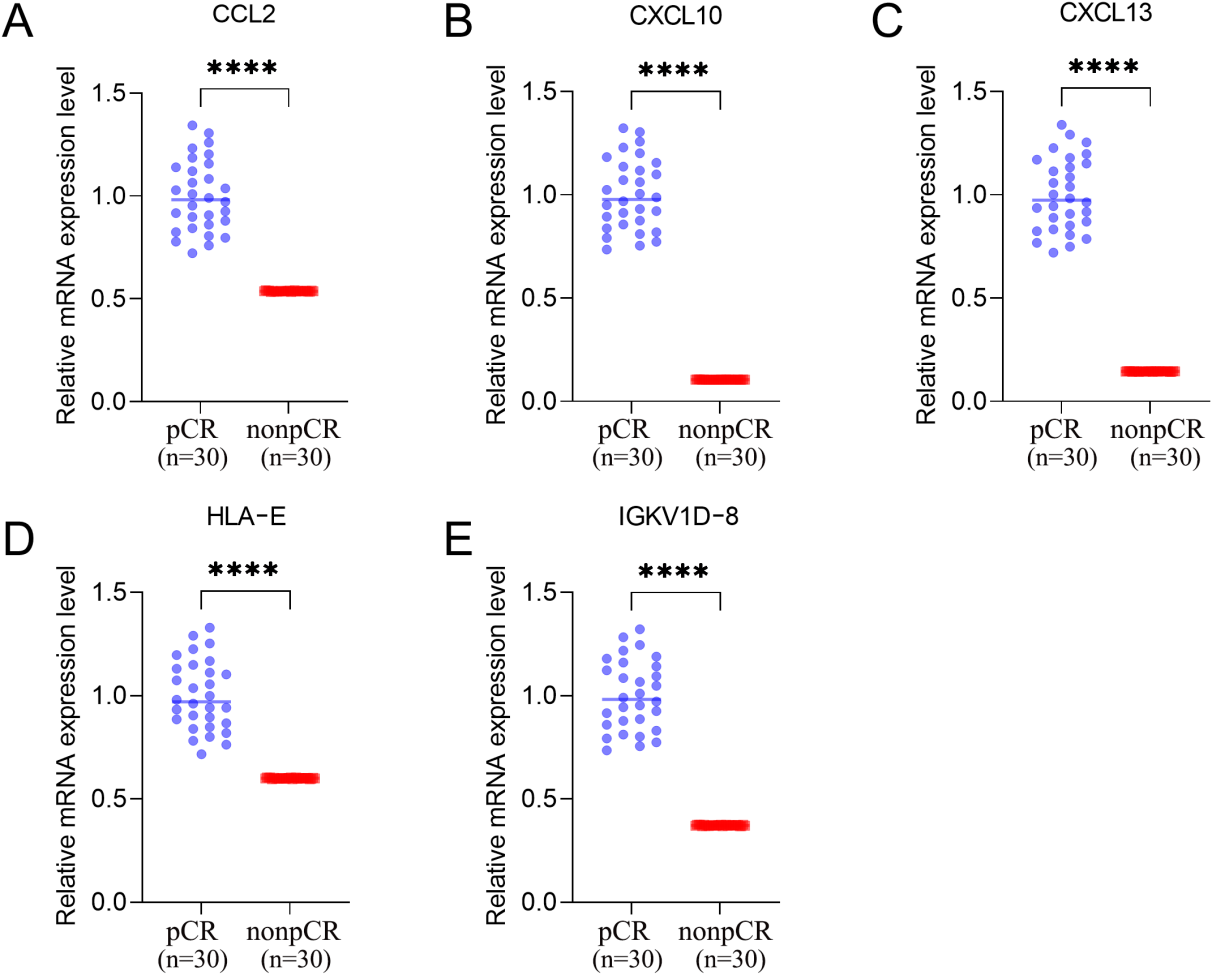
Validation of key genes by qRT-PCR in pretreatment HER2 negative breast tumor tissues. Relative mRNA expression levels of **(A)** CCL2, **(B)** CXCL10, **(C)** CXCL13, **(D)** HLA-E, and **(E)** IGKV1D-8 were significantly higher in patients who achieved pathologic complete response (pCR) compared to non-pCR cases. **** indicates p < 0.0001.

To experimentally validate the five identified immune biomarkers, we measured their mRNA expression levels in pretreatment breast tumor tissues from patients with and without pCR. qRT-PCR analysis demonstrated that CCL2, CXCL10, CXCL13, HLA-E, and IGKV1D-8 were all significantly upregulated in pCR cases compared to non-pCR cases (p < 0.0001; **Figures 10A–E**). These results were consistent with prior transcriptomic findings from the GSE194040 cohort and support the predictive value of these immune genes in determining neoadjuvant chemotherapy response.

## 4 Discussion

This study provides a systematically established and validated ANN-driven immune predictor for neoadjuvant chemotherapy efficacy in HER2-negative disease. We developed and validated an immune-related predictive model based on an ANN to estimate the likelihood of pathologic complete response (pCR) before treatment. We further analyzed transcription factors, constructed a ceRNA network, and performed drug enrichment to identify potential immunotherapeutic targets. By integrating ten independent GEO transcriptomic cohorts, we included 2,385 HER2-negative patients treated with anthracycline and taxane regimens and identified immune gene features closely associated with chemosensitivity.

Fang et al. identified nine immune genes and built a model in a single cohort of 159 patients with an AUC near 0.84 but without external validation (14). Other studies in early HR-positive and HER2-negative breast cancer linked immune effector infiltration and interferon-related gene upregulation to better prognosis and greater sensitivity to systemic therapy, yet sample sizes were limited and most work focused on prognostic stratification rather than practical models for predicting neoadjuvant response with external validation (29). Our data sources covered independent cohorts from different regions and populations and showed consistent performance under strict external testing, which reduces overfitting and cohort bias. Cross-cohort integration and validation are recommended principles in biomarker studies and improve reliability (30). Chen et al. used immune-related genes to build a machine-learning model for predicting response to neoadjuvant chemotherapy in breast cancer and reached an AUC of about 0.75 in two external cohorts (31). Our study combined multiple machine learning algorithms to capture robust associations between immune genes and response. Five key immune-related genes, namely CCL2, CXCL10, CXCL13, HLA-E, and IGKV1D-8 were identified. These genes showed higher expression in the pCR group, and qRT-PCR on our clinical tumor specimens confirmed consistent mRNA upregulation. Immune infiltration, pathway enrichment, transcriptional regulation, and drug association analyses showed that the model’s key genes tracked with an activated tumor immune state. ANN was trained to learn potential nonlinear relationships among features, which improved sensitivity and specificity, underscoring the potential of deep learning for multigene prediction in oncology.

CCL2 is a key chemokine that recruits monocytes and macrophages and participates in antigen presentation and inflammatory infiltration, thereby enhancing recognition by effector cells (32–34). It was higher in the pCR group in our data, suggesting a role in strengthening immune-mediated tumor killing. CXCL10 is an interferon-inducible chemokine that attracts Th1-type CD8 T cells and NK cells through CXCR3 and promotes cytotoxic responses (35). Elevated CXCL10 has been linked to increased TILs and higher pCR rates in breast cancer and is consistent with our findings (36, 37). CXCL13, mainly produced by follicular helper T cells, recruits B cells and supports tertiary lymphoid structure formation, a hallmark of active anti-tumor immunity (38). We observed higher CXCL13 in the pCR group, indicating enhanced B and T cell coordination. HLA-E is a non-classical MHC class I molecule involved in NK-cell regulation (39). Although traditionally considered a mediator of immune escape, recent work shows HLA-E upregulation can accompany interferon pathway activation and reflect ongoing immune responses, a pattern that was evident in the pCR group in our analysis (40). IGKV1D-8 encodes a kappa light chain variable region and indicates active B-cell or plasma-cell infiltration when highly expressed, consistent with beneficial humoral immunity in ER-negative disease (41–43). qRT-PCR on pretreatment tumor tissues confirmed significantly higher mRNA levels of all five genes in pCR cases than in non-pCR cases, echoing the multi-cohort GEO results and supporting biological relevance and clinical reproducibility.

Immune infiltration analysis showed enrichment of effector populations in pCR tumors, including CD8 cytotoxic T cells, activated memory CD4 T cells, follicular helper T cells, M1 macrophages, and activated dendritic cells. Non-pCR tumors were enriched for regulatory T cells, M2 macrophages, and resting plasma cells, showing an immunosuppressive state. These differences between immune-hot and immune-cold tumors are consistent with prior evidence relating TILs to chemotherapy response (44, 45). Correlation networks indicated that CCL2, CXCL10, CXCL13, HLA-E, and IGKV1D-8 were positively associated with CD8 T cells, activated CD4 T cells, and M1 macrophages and negatively associated with M2 macrophages, Tregs, and resting plasma cells, highlighting their role in maintaining a pro-inflammatory, anti-tumor milieu. GSEA showed high-expression groups enriched for antigen processing and presentation, cytokine and receptor interactions, and chemokine signaling. Transcription factor analysis identified NF-κB1, RELA, STAT1, STAT3, and IRF1 as upstream regulators of CCL2 and CXCL10 and IRF4 and SPI1 as regulators of CXCL13. These TFs are central to inflammatory and interferon signaling and are important in tumor immune responses and stress pathways (46, 47). The ceRNA network suggested multi-layered non-coding RNA regulation. LncRNAs such as LINC00969, PRKCQ-AS1, and MUC19 may modulate CXCL13 through miR-125a-5p, miR-1238-3p, and others, which could help explain inter-patient variability in immune activation. Drug enrichment implicated compounds with immunomodulatory or anti-inflammatory activity, including pioglitazone, roflumilast, ibuprofen, and rutin, many linked to PPAR-γ signaling or cytokine regulation. Prior data suggest such agents can influence macrophage polarization and reduce immunosuppressive factors, thereby enhancing anti-tumor immunity (48–50). These findings point to small-molecule immunomodulation as a potential adjunct to increase neoadjuvant sensitivity in HER2-negative disease. This study involved comparisons across 22 immune cell types, which introduces the possibility of inflated type I error. Although false discovery rate correction was applied, some immune cell differences did not retain statistical significance after adjustment and should be interpreted cautiously. Therefore, immune infiltration findings should be viewed as supportive evidence rather than definitive biomarkers, and further validation in independent cohorts and experimental settings is warranted.

A major novelty of this study lies in the combination of large scale multi cohort integration with artificial neural network-based modeling to predict neoadjuvant chemotherapy response specifically in HER2 negative breast cancer. Unlike most previous studies that relied on single datasets or limited sample sizes (51, 52), we integrated ten independent transcriptomic cohorts comprising 2,385 patients treated with anthracycline and taxane based neoadjuvant regimens. This design substantially reduced cohort specific bias and enhanced the robustness and generalizability of the identified immune signature. In addition, we applied an ANN framework to capture nonlinear relationships among immune related genes, which are difficult to model using traditional linear or single algorithm approaches. By combining differential expression analysis, weighted gene co expression network analysis, and multiple machine learning methods based on feature selection strategies, we identified a compact immune gene set that consistently predicted pathologic complete response across independent cohorts.

Several limitations warrant consideration. First, our analyses were primarily based on retrospective GEO datasets, which are inherently subject to potential selection bias and heterogeneity in patient characteristics and treatment details. To mitigate these issues, we applied strict inclusion criteria during cohort selection, focusing on patients who received anthracycline based neoadjuvant chemotherapy, thereby improving treatment regimen homogeneity and reducing confounding effects related to therapeutic variability. Nevertheless, residual heterogeneity in clinical variables, such as patient demographics, tumor burden, and supportive treatment strategies, could not be fully eliminated due to incomplete annotation in public datasets. Therefore, although the multi cohort design and consistent validation support the robustness of our findings, prospective studies with standardized clinical data collection are required to further validate the predictive value and clinical utility of the proposed immune signature. The cohorts analyzed largely reflect chemotherapy alone. With the increasing use of checkpoint inhibitors in the neoadjuvant setting for triple-negative disease, the interaction between chemotherapy and immunity becomes more complex. Whether the present model applies to chemo-immunotherapy requires further evaluation. Further work should focus on validating the proposed immune signature in large scale, prospective, and preferably multicenter cohorts to further assess its robustness and clinical utility. In addition, subtype specific refinement within HER2 negative breast cancer, such as stratification by hormone receptor status or intrinsic molecular subtypes, may improve predictive accuracy. Integration of additional omics layers, including proteomics or spatial immune profiling, could provide complementary biological insights and facilitate the translation of this signature toward clinically actionable biomarkers.

## 5 Conclusion

This study developed and validated a robust immune-related predictive model based on an artificial neural network (ANN) to estimate the response to anthracycline- and taxane-based neoadjuvant chemotherapy in HER2-negative breast cancer. By integrating ten independent GEO cohorts and applying multi-algorithm feature selection, we identified five key immune genes (CCL2, CXCL10, CXCL13, HLA-E, and IGKV1D-8) that define an immune-activated tumor microenvironment associated with higher pCR rates. The ANN model achieved excellent discrimination and generalization across multi-cohort datasets, outperforming traditional statistical models. Immune infiltration, transcription factor, ceRNA, and drug enrichment analyses further demonstrated that these genes participate in pro-inflammatory and cytotoxic immune pathways, providing insight into the immunologic basis of chemotherapy sensitivity. This work establishes an effective and generalizable framework for immune biomarker–driven prediction in HER2-negative breast cancer and highlights the potential of AI-assisted modeling to guide individualized neoadjuvant treatment strategies and identify novel targets for immune modulation.

## Data Availability

The original contributions presented in the study are included in the article/supplementary material. Further inquiries can be directed to the corresponding authors.
